# Administration of intrapulmonary sodium polyacrylate to induce lung injury for the development of a porcine model of early acute respiratory distress syndrome

**DOI:** 10.1186/2197-425X-2-5

**Published:** 2014-02-26

**Authors:** William R Henderson, Julian Barnbrook, Paolo B Dominelli, Donald EG Griesdale, Tara Arndt, Yannick Molgat-Seon, Glen Foster, Gareth L Ackland, James Xu, Najib T Ayas, Andrew W Sheel

**Affiliations:** Division of Critical Care Medicine, Department of Medicine, Faculty of Medicine, University of British Columbia, Vancouver, British Columbia V6T 1Z4 Canada; School of Kinesiology, University of British Columbia, Vancouver, British Columbia V6T 1Z1 Canada; Centre for Comparative Medicine, University of British Columbia, Vancouver, British Columbia V6T 1Z3 Canada; School of Health and Exercise Sciences, University of British Columbia Okanagan, Kelowna, British Columbia V1V 1V7 Canada; Wolfson Institute for Biomedical Research, Department of Medicine, University College London, London, WC1E 6BT UK; Undergraduate Medical Program, Faculty of Medicine, University of British Columbia, Vancouver, British Columbia V6T 1Z3 Canada; Division of Critical Care Medicine, Vancouver General Hospital, ICU2, JPPN 2nd Floor, Room 2438, 855 West 12th Avenue, Vancouver, British Columbia V5Z 1 M9 Canada

**Keywords:** Adult respiratory distress syndrome, Hemodynamics, Respiration, Pulmonary alveoli

## Abstract

**Background:**

The loss of alveolar epithelial and endothelial integrity is a central component in acute respiratory distress syndrome (ARDS); however, experimental models investigating the mechanisms of epithelial injury are lacking. The purpose of the present study was to design and develop an experimental porcine model of ARDS by inducing lung injury with intrapulmonary administration of sodium polyacrylate (SPA).

**Methods:**

The present study was performed at the Centre for Comparative Medicine, University of British Columbia, Vancouver, British Columbia. Human alveolar epithelial cells were cultured with several different concentrations of SPA; a bioluminescence technique was used to assess cell death associated with each concentration. In the anesthetized pig model (female Yorkshire X pigs (*n* = 14)), lung injury was caused in 11 animals (SPA group) by injecting sequential aliquots (5 mL) of 1% SPA gel in aqueous solution into the distal airway via a rubber catheter through an endotracheal tube. The SPA was dispersed throughout the lungs by manual bag ventilation. Three control animals (CON group) underwent all experimental procedures and measurements with the exception of SPA administration.

**Results:**

The mean (± SD) ATP concentration after incubation of human alveolar epithelial cells with 0.1% SPA (0.92 ± 0.27 μM/well) was approximately 15% of the value found for the background control (6.30 ± 0.37 μM/well; *p* < 0.001). Elastance of the respiratory system (*E*_RS_) and the lung (*E*_L_) increased in SPA-treated animals after injury (*p* = 0.003 and *p* < 0.001, respectively). Chest wall elastance (*E*_CW_) did not change in SPA-treated animals. There were no differences in *E*_RS,_*E*_L,_ or *E*_CW_ in the CON group when pre- and post-injury values were compared. Analysis of bronchoalveolar lavage fluid showed a significant shift toward neutrophil predominance from before to after injury in SPA-treated animals (*p* < 0.001) but not in the CON group (*p* = 0.38). Necropsy revealed marked consolidation and congestion of the dorsal lung lobes in SPA-treated animals, with light-microscopy evidence of bronchiolar and alveolar spaces filled with neutrophilic infiltrate, proteinaceous debris, and fibrin deposition. These findings were absent in animals in the CON group. Electron microscopy of lung tissue from SPA-treated animals revealed injury to the alveolar epithelium and basement membranes, including intra-alveolar neutrophils and fibrin on the alveolar surface and intravascular fibrin (microthrombosis).

**Conclusions:**

In this particular porcine model, the nonimmunogenic polymer SPA caused a rapid exudative lung injury. This model may be useful to study ARDS caused by epithelial injury and inflammation.

## Background

Acute respiratory distress syndrome (ARDS) is characterized by severe hypoxic respiratory failure and is associated with high mortality and morbidity [1]. Despite advances in the understanding and treatment of ARDS, the mechanisms of alveolar epithelial injury are not well understood. Loss of alveolar epithelial and endothelial integrity causes a progressive influx of protein-rich fluid into the alveoli, impairs transepithelial fluid transport, and inhibits reabsorption of alveolar edema [2–4]. The presence of surface-active and water-soluble agents in the alveoli contributes to the inactivation of surfactant [5–7]. In ARDS, surfactant depletion and inactivation decreases alveolar stability, exacerbating or precipitating alveolar collapse, lung atelectasis, and hypoxia. Animal models are widely used to study the pathogenic mechanisms of ARDS and to assess the effects of interventions on clinical and biological outcomes [8, 9]. It is recognized that different models emphasize different aspect of ARDS and that no model fully replicates the histological findings characteristic of human ARDS (i.e., inflammatory infiltrates, thickened alveolar septae, intravascular microthrombosis, and hyaline membrane deposition). Therefore, there is a need to create animal models that more accurately mimic the histopathological changes apparent in human ARDS [9]. A guideline committee of the American Thoracic Society (ATS) concluded that a high-quality model of experimental ARDS should include ‘very relevant’ evidence of at least three of four criteria: tissue injury, alteration of the alveolar-capillary barrier, presence of an inflammatory response, and evidence of physiological dysfunction [9].

ARDS may be broadly characterized as being of pulmonary or extrapulmonary origin, with the primary pathological lesion involving a direct insult to the alveoli (such as pneumonia) or an indirect insult to the lung parenchyma and pulmonary endothelial damage from an extrapulmonary disease such as sepsis [10, 11]. There is a substantial body of research suggesting that the morphology and responses to clinical interventions differ between these two sub-groups of ARDS [12–19]. Similarly, experimental models of ARDS may be separated into those that target the alveolar epithelium (analogous to ‘pulmonary’ ARDS) and those that target the vascular endothelium (i.e., ‘extrapulmonary’ ARDS).

Sodium polyacrylate (SPA) is an anionic, osmotically active hydrophilic polymer known to absorb 250 times its weight in water [20]. We hypothesized that intrapulmonary administration of SPA would lead to direct injury of alveolar epithelial cells, which could be used as an experimental model that meets the ATS guidelines criteria by causing (i) accumulation of neutrophils and proteinaceous debris in the alveolar or the interstitial space, (ii) evidence of interstitial and intra-alveolar edema, (iii) an increase in the absolute number of neutrophils in bronchoalveolar lavage fluid, and (iv) hypoxemia. Additionally, we aimed to create a model with stable hemodynamics that reflected the histological and pulmonary mechanical alterations characteristic of ARDS of pulmonary rather than extrapulmonary origin [11].

## Methods

### *In vitro* SPA-induced alveolar epithelial cell injury

After being warmed for 15 min in a 70°C water bath, 0.5 mL of sterile 1% SPA gel was placed in 4.5 mL of acellular standard media (Complete Small Airway Epithelial Cell Growth Media; PromoCell GmbH, Heidelberg, Germany) and was mixed by vortexing; this mixture was labeled ‘0.1% SPA’. Using similar methods, mixtures with SPA concentrations of 0.03%, 0.01%, 0.003%, 0.001%, and 0.0003% were created. One milliliter of cryopreserved human lung epithelial cells (PromoCell lot 2102203, PromoCell GmbH, Heidelberg, Germany) was expanded in standard media. Cell viability was confirmed to be acceptable using a 7-aminoactinomycin D dye exclusion assay (BD Via-Probe, BD Biosciences, San Jose, CA, USA) and flow cytometry (Accuri C6, BD Biosciences, San Jose, CA, USA). The number of cells per milliliter in the prepared suspension was evaluated using a cell counter (Coulter-Z, Beckman Coulter, Inc., Indianapolis, IN, USA). The cell suspension was further diluted as required using standard media to prepare a stock cell suspension of 50,000 cells/mL. Aliquots (100 μL) of the diluted cell suspension were added to the wells of a 96-well plate to obtain a final concentration of 5,000 cells/well. Aliquots (100 μL) of the appropriate SPA concentrations were dispensed to create eight replicates of each dose concentration. Eight control wells held cells and did not receive any SPA. Plated cells were incubated for 2 days. For each plate, 100 μL of ATP enumeration reagent (Hemogenix, Colorado Springs, CO, USA) was then added to each well to lyse the cells, which would release ATP and produce bioluminescence [21]. The bioluminescence emitted was detected and measured using a plate luminometer (Spectramax L, Molecular Devices, Downingtown, PA, USA) in relative luminescence units (RLU). Using an ATP standard curve, sample RLUs were converted to molar units of ATP and a dose–response curve was generated.

### *In vivo* experimental arrangement

#### Animals

Fourteen adult female Yorkshire X pigs were quarantined for 1 week before the experimental sessions. Eleven animals were subjected to experimental lung injury induced by SPA (SPA group). Three pigs served as controls and underwent all interventions and tests with the exception of SPA administration (CON group). All experiments were approved by the Animal Research Committee of the University of British Columbia, Vancouver, British Columbia (certificate no. A11-0396) and conformed to the policies and guidelines of the Canadian Council on Animal Care as well as the ethical standards laid down in the 1964 Declaration of Helsinki and its later amendments.

**Figure 1:**
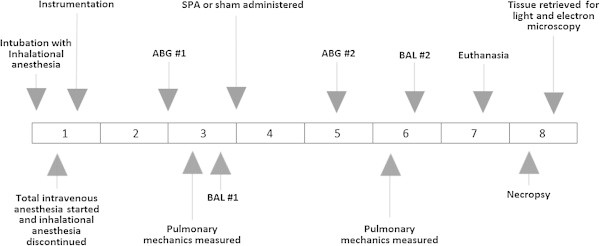
**Schematic timeline of procedures and data collection during the study.** Horizontal numbers in the center of the figure represent time from initial anesthetic. ABG, arterial blood gas; BAL, brochoalveolar lavage; SPA, sodium polyacrylate.

The timelines of the experimental procedures are depicted schematically in Figure 1. After sedation (telazol, 4 to 6 mg/kg (intramuscular)), the animals were placed in the supine position and anesthesia was induced by inhalation of isoflurane (3% to 5% in oxygen). The trachea was intubated using an endotracheal tube (8 or 9 mm diameter). Anesthesia was maintained with isoflurane inhalation (end-tidal concentration 2% to 2.5% in oxygen) until intravenous anesthesia was established (midazolam, 0.1 mg/kg intravenous bolus, followed by propofol infusion (starting at 200 μg/kg/min then adjusted to 150 to 300 μg/kg/min according to depth of anesthesia)). Mechanical ventilation (Puritan-Bennett 7200, Covidien, Ireland) was maintained with the following parameters: positive end-expiratory pressure (PEEP), 0 cm H_2_O; fraction of inspired oxygen (FIO_2_), 0.5; and tidal volume (V_T_), 12 mL/kg of body weight with constant inspiratory flows. These parameters were selected to mimic V_T_ and PEEP noted in similar investigations of experimental ARDS in pigs [22–24]. The respiratory rate was 15 breaths/min initially and was adjusted to maintain end-tidal carbon dioxide partial pressure (PetCO_2_) within 35 to 45 mmHg.

A catheter was placed in the right femoral artery to enable continuous blood pressure measurement (calibrated at the level of the heart) and collection of arterial blood samples. A pulmonary artery catheter was placed percutaneously in the right jugular vein to enable measurement of cardiac output and pulmonary vascular hemodynamic parameters. A urinary catheter and rectal temperature probe were placed. Core temperature was maintained between 35.5°C and 36.5°C using a heated operating table. Mean arterial blood pressure was maintained at >65 mmHg by infusion of phenylephrine (0.1 to 0.3 μg/kg/min) when required.

Before all measurements of pulmonary mechanical parameters, a midazolam bolus (0.1 mg/kg intravenously) was administered and neuromuscular blockade was induced with pancuronium (0.05 to 0.1 mg/kg intravenously) or rocuronium (1 mg/kg intravenously) and monitored by assessment of response to train-of-four stimulation using a peripheral nerve stimulator. The adequacy of sedation and analgesia were assessed using physical examination and vital signs taking at 30-min intervals by experienced animal care technicians.

Lung injury in the SPA group was induced by injecting sequential aliquots (5 mL) of 1% SPA gel in aqueous solution into the distal airway with a rubber catheter through the endotracheal tube. The SPA was dispersed throughout the lungs by manual bag ventilation (approximately 200 mL/breath). Aliquots of SPA were administered approximately every 10 min until hypoxemia was achieved, as defined by arterial partial pressure of oxygen (PaO_2_) of <150 mmHg with FIO_2_ = 0.5. Control animals underwent placement of a red rubber catheter through the endotracheal tube and underwent manual bagging (approximately 200 mL/breath) but received no intratracheal SPA.

### Pulmonary mechanics, oxygenation, and hemodynamics

Inspiratory and expiratory flow rates were measured using a pneumotachograph (model 3813, Hans Rudolph, Kansas City, MO, USA) that was calibrated using a 2-L syringe. Volumes were obtained by numerical integration of the flow signals. Mouth pressure was measured at a port placed between the ventilator wye and the endotracheal tube. Esophageal pressure was measured using a balloon-tipped catheter (no. 47–9005, Ackrad Laboratory, Cranford, NJ, USA) placed in the lower one third of the esophagus. Catheter position was deemed satisfactory when the change in esophageal pressure was roughly equal to the change in airway pressure during passive inspiration [25]. Esophageal and airway pressures were both measured using calibrated piezoelectric pressure transducers (Raytech Instruments, Vancouver, British Columbia, Canada). Dynamic and static pulmonary mechanical variables were calculated as previously described [26, 27]. Functional residual capacity (FRC) was measured using a previously described helium dilution method to assess lung recruitment before and after injury [28]. Arterial blood gases were measured immediately before and 1 h after lung injury. Arterial blood pressure, pulmonary artery pressure, and heart rate were continuously monitored using surface electrocardiography. All data were collected and recorded using an integrated 16-channel data acquisition and recording system (PowerLab/16SP model ML 795 and LabChart v7, ADI, Colorado Springs, CO, USA). Cardiac output was measured using thermodilution curves generated from injection of 5 mL boluses of cold 0.9% saline solution that were analyzed using the same software. Three bolus injections were performed sequentially once pulmonary artery temperature returned to baseline, with the average reported.

All animals were euthanized at the end of the experiment using an intravenous bolus of pentobarbital sodium (120 mg/kg). Death was confirmed by the absence of cardiac electrical activity on continuous surface electrocardiography.

### Gross and microscopic pathology

All animals underwent bronchoalveolar lavage (BAL) immediately before SPA-induced lung injury or sham injury and again before euthanization using previously described procedures [29, 30]. Direct cellular counts were performed on aliquots of BAL fluid using an automated cell counter. Direct and cytocentrifuge smears were prepared for cytological examination using modified Wright-Giemsa stain. The white blood cell (WBC) and differential counts were performed with 100 to 300 cells and the percentages were determined.

The samples of the lung tissue from SPA-treated animals were excised from the dorsal-caudal regions of both lungs and fixed in 10% buffered formalin for 48 h before trimming. Similar samples were retrieved from control animals. Fixed tissues were processed (Tissue-Tek VIP 5 vacuum infiltration tissue processor, Sakura Finetek USA, Torrance, CA, USA), embedded in paraffin, sectioned (thickness 4 μm), and stained with hematoxylin-eosin. For each animal, ten randomly selected fields were assessed (original magnification × 400) for alveolar fibrin deposition, alveolar inflammatory cell infiltration, and interstitial and intra-alveolar edema [31].

Electron microscopy was performed on post-mortem lung tissue. To accomplish this, after euthanization, the lungs were flushed with glucose-containing Krebs buffer. After perfusion with 2.5% glutaraldehyde buffered in 0.1 M sodium cacodylate, the lungs were excised and sampled for damage. The excised tissue was cut into smaller pieces (1 mm^3^) and further fixed in 2.5% glutaraldehyde in 0.1 M sodium cacodylate buffer for 24 h. The tissue was washed with 0.1 M sodium cacodylate buffer, post-fixed in a mixture of 2% potassium ferrocyanate and 2% osmium tetroxide in 0.1 M sodium cacodylate, dehydrated in a graded series of acetone solutions, infiltrated, and embedded. The sections (thickness 500 nm) were cut (Leica EM UC6 microtome, Leica Microsystems, Wetzlar, Germany) and viewed under light microscopy. Sections (thickness 60 nm) were stained with saturated uranyl acetate and lead citrate and examined using a transmission electron microscope (Tecnai 12, FEI, Hillsboro, OR, USA) at original magnification × 1,850 to × 9,700.

### Data analysis

Data were reported as mean ± SD, unless otherwise indicated. Continuous variables were analyzed using paired *t* tests (within subject) or independent *t* tests (between subjects) where appropriate. All tests were two-sided and statistical significance was defined at *p* < 0.05. Statistical analyses were performed using STATA 10.0 Statistical Software (StataCorp, College Station, TX, USA).

## Results

### *In vitro* SPA-induced alveolar epithelial cell injury

**Figure 2:**
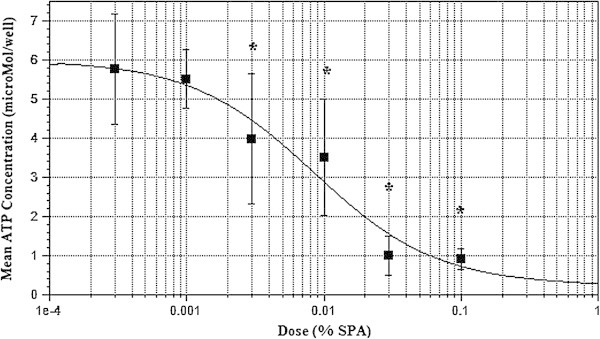
**Mean concentration of ATP found in wells of human alveolar cells after incubation with SPA.** Asterisk represents *p* < 0.05 for values compared with baseline.

Data were obtained from all wells of human alveolar cells cultured with and without SPA. The mean ATP concentration after incubation with 0.1% SPA (0.92 ± 0.27 μM/well) was approximately 15% of that found in control wells (6.30 ± 0.37 μM/well; *p* < 0.001). The addition of progressively higher doses of SPA decreased the viability of human alveolar epithelial cells, indicated by absolute concentration (μM/well) of ATP and as a percentage of the control ATP response (Figure 2). The effect of SPA on cell culture survival exhibited a dose–response curve best described as a reverse sigmoidal function with an *R*^2^ value of 0.96. The SPA concentration that inhibited cell survival in 50% of cells (IC_50_) in the preparation used was estimated to be 0.008%.

### *In vivo* experiments

The 14 pigs used in the present study had a mean body weight of 29.5 ± 4.5 kg. The SPA group received 15.5 ± 4.2 mL SPA (0.54 ± 0.18 mL/kg intratracheally) while the CON group received none. SPA pigs were ventilated at V_T_ of 12.53 ± 1.34 mL/kg and CON pigs with a V_T_ of 13.68 ±1.91 mL/kg (p = 0.25).

### Pulmonary mechanics, oxygenation, and hemodynamics

All pulmonary mechanical data are presented in Table 1. Elastances of the respiratory system (*E*_RS_) and of the lung (*E*_L_) were significantly higher after injury compared with before injury in SPA-treated animals. *E*_RS_ was greater in SPA-treated animals than in the CON group after injury, and increases in *E*_RS_ in the SPA group were driven exclusively by changes in *E*_L_ because *E*_CW_ did not increase after injury. There were no differences in *E*_RS_, *E*_L,_ or *E*_CW_ in CON animals when pre-injury and post-injury values were compared. *E*_L_ was not significantly higher in SPA than CON after injury. Given the significant changes between uninjured and injured states in SPA, as well as the differences in *E*_CW_ between SPA and CON after injury, this lack of difference likely reflects the small sample size for the CON group. While there were no differences in measures of resistance between the SPA and CON groups before injury, respiratory system resistance (*R*_RS_) more than doubled after injury in the SPA group and appeared to be driven by increases in lung resistance (*R*_L_). Chest wall resistance (*R*_CW_) did not change from before to after injury in SPA-treated animals, and measures of resistance did not change significantly in the CON group when before and after injury data were compared.

**Table 1:** **Pulmonary mechanics of animals before and after real injury with SPA or sham injury (CON)**

	SPA group before injury	CON group before injury	***p*** value SPA versus CON before injury	SPA group after injury	CON group after injury	***p*** value SPA versus CON after injury	***p*** value SPA before versus after injury	***p*** value CON before versus after injury
*E*_RS_ (cm H_2_O/L)	33.56 ± 10.03	31.88 ± 2.53	0.77	65.08 ± 29.78	32.88 ± 13.72	0.04	<0.01	0.99
*E*_L_ (cm H_2_O/L)	21.06 ± 8.34	19.13 ± 2.15	0.76	56.87 ± 25.07	23.68 ± 13.82	0.11	<0.01	0.70
*E*_CW_ (cm H_2_O/L)	15.98 ± 8.32	13.15 ± 5.51	0.66	18.24 ± 19.01	11.52 ± 4.72	0.64	0.73	0.79
*R*_RS_ (cm H_2_O/L/s)	10.66 ± 6.83	15.26 ± 6.43	0.31	21.55 ± 10.99	12.99 ± 2.15	0.22	0.01	0.60
*R*_L_ (cm H_2_O/L/s)	9.59 ± 6.55	16.17 ± 8.05	0.22	21.00 ± 9.61	13.18 ± 2.07	0.29	<0.01	0.67
*R*_CW_ (cm H_2_O/L/s)	1.58 ± 1.15	1.21 ± 0.66	0.68	2.48 ± 3.55	0.93 ± 0.75	0.57	0.43	0.73

*E*_RS_, elastance of the respiratory system; *E*_L_, elastance of the lung; *E*_CW_, elastance of the chest wall; *R*_RS,_ resistance of the respiratory system; *R*_L_, resistance of the lung; R_CW_, resistance of the chest wall. All values are reported as mean ± standard deviation.

FRC decreased from 16.4 ± 3.2 mL/kg before injury to 10.7 ± 3.3 mL/kg after injury in SPA-treated animals (*p* < 0.001) and was unchanged in CON animals, with values before and after sham injury of 13.8 ± 2.2 and 12.77 ± 1.52 mL/kg, respectively (*p* = 0.54). PaO_2_ decreased in SPA-treated animals with injury from 224.6 ± 48.4 to 72.27 ± 12.51 mmHg (*p* < 0.01) and was unchanged in CON animals, with values before and after sham injury of 250.67 ± 108.61 and 244.67 ± 27.14 mmHg, respectively (*p* = 0.93). PaO_2_ values were not different between SPA and CON animals before injury (*p* = 0.53) but were different after injury (*p* < 0.01).

All hemodynamic data are presented in Table 2. As expected, cardiac output and mean arterial pressure did not change before and after injury in SPA-treated and CON animals. Mean pulmonary arterial pressures (MPAP) in SPA and CON animals were not different before injury (*p* = 0.29). MPAP increased in SPA-treated animals with injury (*p* < 0.01), but not in CON animals (*p* = 0.07). No animals required vasopressor support before injury. Following injury, 2 of 11 (18%) SPA animals but none of the CON animals required vasopressors (*p* = 1.0).

**Table 2:** **Selected hemodynamic parameters of animals before and after injury with SPA or sham injury (CON)**

	SPA group before Injury	CON group before injury	***p*** value SPA versus CON before injury	SPA group after injury	CON group after injury	***p*** value SPA versus CON after injury
MAP (mmHg)	82.96 ± 8.88	86.4 ± 4.36	0.54	79.84 ± 15.58	88.8 ± 8.64	0.37
CO (ml/kg)	50.31 ± 8.29	43 ± 13.74	0.35	49.72 ± 7.25	35.55 ± 4.59	0.26
MPAP (mmHg)	20.96 ± 4.06	18 ± 4.25	0.29	34.83 ± 5.95	26.27 ± 4.2	0.04
Subjects requiring vasopressors (*n*)	0	0	1.0	2	0	1.0

MAP, mean arterial pressure; CO, cardiac output; MPAP, mean pulmonary arterial pressure. All values are reported as mean ± standard deviation.

### Gross and microscopic pathology

BAL fluid retrieved before injury revealed WBC differential counts that were similar between SPA and CON animals and were within the acceptable reference intervals [29–32]. BAL fluid retrieved from SPA-treated animals after injury demonstrated an increase in WBC counts and neutrophilia, while BAL fluid from CON animals after sham injury demonstrated no changes from pre-injury values. The difference in BAL red blood cell (RBC) concentrations between CON and SPA animals noted prior to injury did not persist after injury. While the cause of this is unclear, it is likely to be of limited significance. Detailed results from BAL samples are summarized in Table 3.

**Table 3:** **Bronchoalveolar lavage results of animals before and after injury with SPA or sham injury (CON)**

	SPA group before injury	CON group before injury	***p*** value SPA versus CON before injury	SPA group after injury	CON group after injury	***p*** value SPA versus CON after injury
RBC × 10^12^/L	0.01 ± 0.01	0.10 ± 0.00	<0.01	0.05 ± 0.10	0.02 ± 0.01	0.53
WBC × 10^9^/L	0.40 ± 0.22	0.61 ± 0.39	0.25	3.20 ± 1.63	0.36 ± 0.21	0.02
Macrophages (% of WBC)	65.89 ± 22.21	54.00 ± 16.37	0.42	5.56 ± 5.73	48.33 ± 14.43	<0.01
Small lymphocytes (% of WBC)	23.78 ± 18.85	23.33 ± 5.77	0.97	2.33 ± 0.71	31.67 ± 12.58	<0.01
Neutrophils (% of WBC)	10.33 ± 14.00	22.67 ± 16.17	0.23	91.44 ± 5.48	26.67 ± 20.21	<0.01

RBC, red blood cells; WBC, white blood cells. All values are reported as mean ± standard deviation.

**Figure 3:**
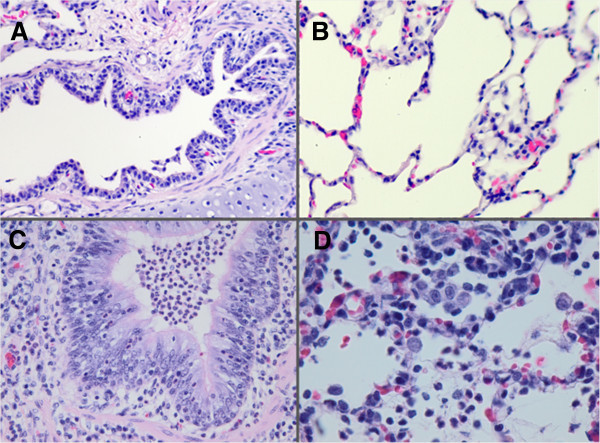
**Histology of lung tissue from CON and SPA-treated animals.** Histology of lung tissue from control (CON) **(A, B)** and sodium polyacrylate (SPA)-treated **(C, D)** animals 6 h after administration of SPA or sham. **(A)** Bronchiole from CON animal demonstrating normal epithelial structure (hematoxylin-eosin stain, original magnification × 20). **(B)** Normal alveolar structure in CON animal (hematoxylin-eosin, original magnification × 40). **(C)** Bronchiole from SPA-treated animal filled with degenerated and viable neutrophils, with transmigration of neutrophils across the respiratory epithelium. Cilia are intact and mild epithelial hyperplasia is present. The subjacent alveoli contain inflammatory cells and fibrin (hematoxylin-eosin stain, original magnification × 20). **(D)** Macrophages, neutrophils, and fibrin strands in an area of alveolar inflammation and consolidation in tissue from an SPA-treated animal (hematoxylin-eosin stain, original magnification × 40).

**Figure 4:**
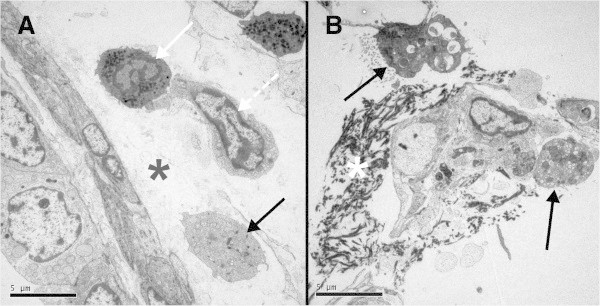
**Transmission electron microscopy of lung tissue from pig subjected to experimental injury with intratracheal SPA. (A)** Interstitium and adventitia of a terminal bronchus with migrating polymorphonuclear leukocyte (solid white arrow), macrophage (dashed white arrow), and monocyte/macrophage (solid black arrow). Edematous exudate (asterisk) is noted (original magnification × 3,900). **(B)** Alveoli with type 2 pneumocytes (black arrows) and dense fibrin deposition (white asterisk) (original magnification × 3,900).

**Figure 5:**
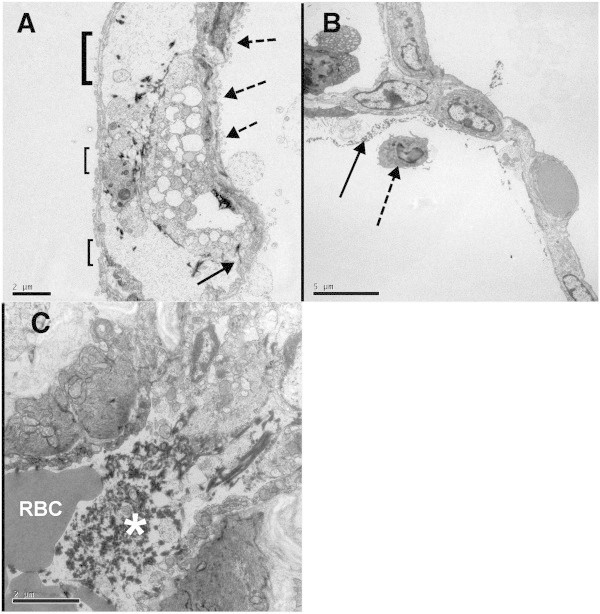
**Transmission electron microscopy of lung tissue from pig subjects to experimental injury with intratracheal SPA. (A)** Alveolar-capillary interface with gaps in the capillary epithelium (open brackets denoting size of gap), necrosis of alveolar epithelium (dashed arrows), and gap in endothelium (solid arrow) (original magnification × 5,800). **(B)** Alveolus with fibrin deposition (solid arrow) and a neutrophil in the air space (dashed arrow) (original magnification × 3,900). **(C)** Perialveolar blood vessel containing fibrin (white asterisk) and a red blood cell (RBC) (original magnification × 9,700).

At necropsy, the lungs from CON animals appeared to be normal. Light microscopy examination of the lungs from CON animals revealed normal-appearing lungs with regions of atelectasis. There was no significant active inflammation in the lungs (Figure 3A,B). At necropsy, the dorsal lungs from SPA-treated animals exhibited marked consolidation and congestion (approximately 45% of the total lung tissue). The cut surfaces of the affected tissues were congested, hemorrhagic, and edematous. Light microscopy of lung specimens revealed bronchiolar and alveolar spaces filled with neutrophilic infiltrate, proteinaceous debris, and fibrin deposition (Figure 3C,D). Although minimal epithelial damage was noted in the large airways, peribronchiolar and interstitial edema and intravascular congestion were evident. Early epithelial hyperplasia with transmigration or exudation of neutrophils across the bronchiolar wall into the airspace was widespread. Similarly, electron microscopy of the lung tissue from SPA animals revealed injury to the alveolar epithelium and basement membranes, including intra-alveolar neutrophils and fibrin on the alveolar surface and intravascular fibrin (microthrombosis) (Figures 4 and 5).

## Discussion

In our study, intrapulmonary administration of SPA resulted in rapid development of the physiological and histological changes characteristic of early ARDS, including diffuse alveolar damage, necrosis of alveolar epithelial cells, inflammatory cell infiltration, and proteinaceous alveolar and interstitial edema. There currently exists marked variability in the characteristics and quality of experimental models of ARDS. The present model of SPA-induced ARDS satisfied the published ATS criteria for a high-quality model [9], indicated by the following: accumulation of neutrophils and proteinaceous debris in the alveolar or the interstitial space, evidence of interstitial and intra-alveolar edema, an increase in the absolute number of neutrophils in BAL fluid, and hypoxemia.

Both our *in vitro* and *in vivo* data suggest that SPA-induced lung injury represents a model of ARDS of pulmonary origin due to epithelial injury. Our data showed that SPA decreased the viability of cultured human alveolar cells in a dose-dependent fashion. SPA is an intensely hydrophilic anionic polymer that has significant osmotic activity [20, 33]. Previous investigations have demonstrated local and systemic toxicity of variable severity with parenteral, enteral, and intratracheal SPA administration in animals [34, 35]. Given the evidence of alveolar epithelial cytotoxicity and the histological evidence of alveolar inflammation presented here, we cautiously speculate that SPA caused ARDS through direct injury to the alveolar epithelium, potentially through the creation of an osmotic gradient across the alveolar epithelial membrane.

Similarly, our *in vivo* data reflect an injury to the lungs, rather than a systemic injury, and are, therefore, consistent with a model of ARDS of pulmonary origin. In contrast to the CON animals, SPA-injured animals rapidly developed hypoxemia and decreases in FRC. Current clinical definitions for ARDS use the PaO_2_/FiO_2_ ratio (P/F) to categorize ARDS severity as mild (P/F < 300), moderate (P/F < 200), or severe (P/F < 100) [36]. The mean P/F ratio after SPA administration was 144.5 ± 25, and all SPA animals had a P/F ratio that qualified as moderate ARDS. The mean FRC decreased by 36% in the SPA group, a decrease that is consistent with alterations in FRC characteristic of patients with ARDS [37]. The alterations in elastance and resistance in the SPA group were attributable to changes in *E*_L_ and *R*_L_ rather than *E*_CW_ and *R*_CW_, suggesting that the injury was intrinsic to the lung (Table 1). In this context, pulmonary artery pressures increased more in SPA-injured animals than in controls, but there were few systemic hemodynamic changes in either group as quantified by cardiac output, mean arterial pressure, or the need for vasoactive medications.

Our experimental model demonstrated that intrapulmonary administration of SPA caused lung injury consistent with injury characteristic of ARDS of pulmonary origin, possibly due to direct alveolar epithelial injury. Both viral and bacterial pneumonias cause alveolar injury in part through apoptosis and necrosis of alveolar types I and II cells [38, 39]. SPA-induced ARDS may therefore represent a useful experimental model of these clinical causes of ARDS. SPA causes interstitial edema, intra-alveolar fibrin deposition, and a neutrophilic alveolitis, rather than the microvascular congestion and less severe alveolar damage characteristic of extrapulmonary causes of ARDS. It is, therefore, reasonable to compare experimental ARDS induced by SPA with other models that cause a primarily alveolar injury, such as saline lavage, injurious mechanical ventilation, hyperoxia, or acid aspiration.

Variability exists in the characteristics and quality of current experimental models of ARDS; lung injury induced by SPA may provide advantages when attempting to study the role of epithelial injury in ARDS. For example, saline lavage precipitates acute hypoxia due to alveolar collapse but is not associated with a significant change in alveolar-capillary permeability or inflammation and, thus, does not reflect the histology that is generally associated with human ARDS [8, 40, 41]. Injurious mechanical ventilation produces a model of ARDS characterized by hypoxemia and inflammation; however, the presumed mechanism is that of mechanotransduction of injurious forces rather than direct epithelial injury. As such, it represents a useful model of ventilator-associated lung injury in already susceptible lungs, but does not represent a likely mode of injury to previously healthy lungs [40]. Similarly, while hyperoxia causes inflammation and injury in some animal models [42, 43], it is unclear whether it initiates ARDS in humans [8, 44]. Acid aspiration (classically, HCL with a pH of 1.5) approximates a model of ARDS similar to that of SPA, with inflammation and direct injury to alveolar epithelial cells [45–47]. However, humans do not usually aspirate strong acids. Rather, gastric contents are a combination of particulate matter, bacteria, and fluids with a pH considerably greater than 1.5. SPA, which is neutral in terms of pH, may allow insights into direct epithelial injury at normal pH. With respect to pathological findings, the lung injury associated with SPA is similar to that induced by oleic acid [48]. In contradistinction to our model, intravenous oleic acid injection produces an injury that has been widely reported to be associated with decrease in cardiac output and increased hemodynamic instability [49–51]. While two of the eleven animals in the SPA group required small amounts of vasopressors after injury, there was no difference in the cardiac output between SPA and CON animals. The hemodynamic stability generally demonstrated in the SPA-injured animals may allow future investigations to separate the effects of epithelial injury from those of hypotension and hypoperfusion, which are two potential confounders often present in other models. However, given the possibility that SPA-induced ARDS may have a systemic component (as suggested by mild hypotension in two of the SPA-injured animals), further delineation of the systemic effects of SPA-associated ARDS should occur first.

Our model also has several limitations that warrant consideration. First, the short duration of our study precludes the ability to describe the evolution of the injury produced beyond the first few hours. While our model appears to accurately mimic the physiological and histological changes apparent in early ARDS, we did not demonstrate that the injury precipitates the delayed histological findings of ARDS. Second, since our *in vivo* data used a longer time frame (2 days) than did our *in vitro* experiment (several hours), this may weaken the relationship between the two sets of data. Similarly, while it appears probable that these data clarify the potential mechanisms of SPA-induced lung injury, these experimental models do not fully replicate the biochemical or physiological milieu found in porcine or human lungs. Importantly, the concentrations of SPA used in the *in vitro* experiments are an approximation of the concentrations that may have been found locally in alveoli in the *in vivo* experiment. Third, human trials have suggested that V_T_ >6 mL/kg may be harmful to injured lung tissue [1]. The animals in our study were ventilated with larger tidal volumes V_T_ (12.53 ± 1.34 mL/kg in the SPA group) and without PEEP, raising the possibility that the changes observed in our model may have been due to injurious ventilation rather than SPA. However, pigs have significantly lower specific lung elastance than humans and tolerate V_T_ of up to 22 mL/kg without evidence of lung injury [52]. The ventilator settings used in our trial were recently used in a similar trial involving pigs that did not demonstrate the anatomical or histological changes characteristic of ARDS [23]. Our control animals did not demonstrate physiological or histological evidence of alveolar injury after several hours of this ventilation strategy. We, therefore, believe that the injury observed in our model was due to SPA rather than an intrinsically injurious ventilation strategy *per se*. Finally, interspecies differences may mean that the findings from this particular porcine model should only be cautiously applied to human patients.

## Conclusions

We demonstrated that intrapulmonary administration of SPA results in rapid exudative lung injury. Furthermore, our study describes an animal model that may be of value in studying ARDS of pulmonary origin. This model may be useful in clarifying the role of direct alveolar injury in the pathogenesis of ARDS. Further studies are needed to better characterize the mechanisms underlying this model of lung injury and its potential contribution in patients with ARDS.
